# Quality of Life and Aesthetic Satisfaction in Patients Who Underwent the “Commando Operation” with Pectoralis Major Myocutaneus Flap Reconstruction—A Case Series Study

**DOI:** 10.3390/healthcare10091737

**Published:** 2022-09-10

**Authors:** Natalija Ivkovic, Dinko Martinovic, Slavica Kozina, Slaven Lupi-Ferandin, Daria Tokic, Mislav Usljebrka, Marko Kumric, Josko Bozic

**Affiliations:** 1 Department of Otorhinolaryngology, University Hospital of Split, 21000 Split, Croatia; 2 Sleep Medicine Center, University of Split School of Medicine, 21000 Split, Croatia; 3 Department of Maxillofacial Surgery, University Hospital of Split, 21000 Split, Croatia; 4 Department of Psychological Medicine, University of Split School of Medicine, 21000 Split, Croatia; 5 Department of Anesthesiology and Intensive Care, University Hospital of Split, 21000 Split, Croatia; 6 Department of Pathophysiology, University of Split School of Medicine, 21000 Split, Croatia

**Keywords:** head and neck squamous cell carcinoma, commando operation, pectoralis major myocutaneus flap, quality of life, sleep impairment

## Abstract

The “commando operation” is an extensive surgical procedure used to treat patients with oral squamous carcinoma and metastasis in the cervical lymph nodes. While the procedure can be curative, it is also very mutilating, which consequently has a major impact on the patient’s quality of life. Several studies showed that the procedure is associated with loss of certain functions, such as impairments in speech, chewing, swallowing, and loss of taste and appetite. Furthermore, some of these impairments and their degree depend on the reconstruction method. However, the data regarding the functional impairments and aesthetic results in patients who underwent the “commando operation” along with the pectoralis major myocutaneus flap reconstruction are still inconclusive. This study included 34 patients that underwent partial glossectomy, ipsilateral modified radical neck dissection, pectoralis major myocutaneus flap reconstruction, and adjuvant radiotherapy. A structured questionnaire was used to evaluate aesthetical results and functional impairments as well as to grade the level of satisfaction with the functional and aesthetic outcomes both by the patients and by the operator. Most of the patients stated that their speech (*N* = 33; 97%) and salivation (*N* = 32; 94.2%) severely changed after the operation and that they cannot chew (*N* = 33; 97%) and swallow (*N* = 33; 97%) the same as before the operation. Moreover, almost half of the patients (*N* = 16; 47%) reported that they have severe sleep impairments. However, only few of the included patients stated that they sought professional help regarding the speech (*N* = 4; 11.7%), eating (*N* = 5; 14.7%), and sleeping (*N* = 4; 11.7%) disturbances. Additionally, there was a statistically significant difference between the operator and the patients in the subjective assessment of the aesthetic results (*p* = 0.047), as operators gave significantly better grades. Our results imply that this procedure and reconstructive method possibly cause impairments that have an impact on the patients’ wellbeing. Moreover, our outcomes also suggest that patients should be educated and rehabilitated after the “commando operation” since most of them were reluctant to seek professional help regarding their impairments. Lastly, sleep deficiency, which was observed after the procedure, should be further explored.

## 1. Introduction

Head and neck squamous cell carcinoma represents the sixth most common malignancy worldwide, with an estimated 3% of all cancer cases [1]. The most important risk factors considered to significantly contribute to the pathogenesis of the head and neck squamous cell carcinoma are tobacco usage; alcohol consumption; and the infection with a high-risk human papilloma virus, particularly type 16 [2,3,4]. One of the most fatal among the head and neck squamous cell carcinoma is the oral squamous cell carcinoma, which accounts for 90% of all oral malignancies and has an estimated 2–3% death rate of all cancer-related deaths [5,6].

Given the complexity of the disease and the sensitivity of the region, oral squamous cell carcinoma treatment should be multidisciplinary, with the highlight not only on the therapy but also on the supportive aftercare. The possible treatment approach involves surgery, radiotherapy, and chemotherapy, all of which severely reduce quality of life [7,8]. Even though the two latter therapeutical modalities are more conservative and preservative for the patient, in most cases, surgical excision is the best and main option [9,10]. The “commando operation” is an extensive and difficult procedure with a wide range of variations, but almost always consists of some degree of glossectomy, mandibulotomy/mandibulectomy, and block dissection of the cervical lymph nodes [11]. It is a vast composite resection used to eradicate the primary oral cancer as well as the regional metastasis, which are most commonly found in the ipsilateral neck. The tumor approach as well as the en-block removal is accomplished with either the “lip split” mandibulotomy technique that consists of a lower lip and chin incision and a mandibular osteotomy, or with the “pull through” technique in which the tumor is pulled through a new opening in the floor of the mouth together with the dissected neck lymph nodes [12,13]. While the procedure can be curative for the patient, it is also very mutilating, which can later have a major impact on the patient’s quality of life. Due to the consequences of the procedure, patients experience loss of certain functions in individually determined degrees. The most seriously impacted functions are speech, chewing, swallowing, and loss of taste and appetite [14]. Additionally, these patients encounter severe oral dryness, tissue stiffness, and facial disfigurement. However, all of these impairments are a heterogeneous group of head and neck surgical consequences. They depend on the extension of the disease, specific structure involvement, and the reconstruction method used after the excision [11,15]. Nowadays, most reconstructions after the composite resection of the head and neck are mostly conducted using free flaps, a revolutionary method in head and neck surgery [16,17,18]. Even though free flaps have their advantages, pectoralis major myocutaneus flap is still an important reconstructive method due to its simple technical aspects, versatility, and proximity to the oral cavity region [19,20].

In the last few decades, the rising global trend in oncological surgery is moving away from focusing only on the therapy of the cancer and is now emphasizing the need to manage the consequences of the treatment [21,22,23]. The quality of life in cancer patients is a dynamic notion concerning all of their subjective life aspects; their general well-being; and their individual needs, beliefs, attitudes, and values [24]. As aforementioned, it is a dynamic notion that changes with time, disease progression, and treatment, and it plays an important role in a patient’s road to recovery and future return to everyday activities [25]. A study conducted by Campbell et al. investigated the quality of life in patients with oral squamous cell carcinoma after different therapeutical modalities [26]. They showed that patients who underwent primary radiotherapy had a better quality of life than those who underwent surgery and adjuvant radiotherapy. Furthermore, several studies determined that patients who underwent surgery with the free flap reconstruction had better functional and cosmetic outcomes compared to those who underwent pectoralis major myocutaneus flap reconstruction [27,28,29]. However, data regarding the functional impairments and aesthetic results in patients who underwent the “commando operation” and the reconstruction of the defect with the pectoralis major myocutaneus flap are still inconclusive.

Hence, the aim of this study was to investigate the quality of life in patients with oral squamous cell carcinoma who underwent composite resection and reconstruction with pectoralis major myocutaneus flap. The parameters that we aimed to evaluate were the aesthetic appearance as well as the functions such as speech, chewing, swallowing, salivation, sleeping, and mood changes. Moreover, we also aimed to compare the subjective assessment of these impairments between the patients and the operator to evaluate possible discrepancies.

## 2. Materials and Methods

### 2.1. Study Design and Ethical Considerations

This case series study was conducted at the University of Split, School of Medicine. All participants were informed about the procedures and the purpose of the study in a timely manner. Furthermore, they all signed a written informed consent form.

The study was performed according to the ethical principles of the latest Helsinki declaration, and it was approved by the Ethics Committee of the University of Split, School of Medicine (no. 003-08/21-03/0003).

### 2.2. Subjects

The study included 34 patients who underwent the “commando operation” at the Department of Maxillofacial Surgery during the period from 2016 to 2020. All of the patients had oral squamous cell carcinoma as well as regional metastasis in the ipsilateral cervical lymph nodes with the extension to the regions I–V, all of which was confirmed by the cytological and pathohistological analysis as well as with radiological imaging. Moreover, all patients underwent adjuvant radiotherapy after the surgical treatment.

The inclusion criteria were as follows: >1 and <5 years from the “commando operation”; ipsilateral modified radical neck dissection (preservation of n. accessorius); reconstruction of the intraoral excision with the pectoralis major myocutaneus flap. Exclusion criteria were as follows: postoperative complications associated with the pectoralis major myocutaneus flap; segmental mandibular resection; reexcision due to the oral squamous cell carcinoma relapse; contralateral/bilateral neck dissection; confirmed distant metastasis of the oral squamous cell carcinoma; osteonecrosis of the jaw due to radiotherapy; other confirmed malignancy; cerebrovascular and neurological diseases; psychiatric and mental diseases; sleep-related disorders; primary salivary glands diseases; alcohol and drug abuse. Pectoralis major myocutaneus-flap-related complications that were excluded were as follows: partial and total flap necrosis; flap dehiscence; salivary leakage; orocutaneous fistulas; infection.

Since according to the most relevant literature, the last phase of wound healing after closure by primary intention is finished after one year, we included only patients who underwent the commando operation at least 1 year prior to the study onset [30,31]. All participants were subjected to a detailed physical examination, and their medical history was reviewed. Moreover, they were interviewed regarding drug, alcohol, and tobacco consumption. All the “commando operations” were conducted by two experienced maxillofacial surgeons.

### 2.3. Questionnaire

A questionnaire was constructed for this study to investigate the aesthetic appearance as well as the functions such as speech, chewing, salivation, and sleeping. With extensive research of the available literature, we did not find any validated questionnaire that examines these parameters from both the perspective of the patient and the observer. Therefore, a structured questionnaire was constructed by a group of experts. The group involved a clinical psychologist, a maxillofacial surgeon, and a nurse. During the conceptualization of the questionnaire, two parts were distinguished. The first one contained six domains: “speech”, “eating ability”, “salivation”, “sleeping quality”, “mood changes”, and “aesthetic results”. Moreover, the second part had two domains: “aesthetic results” and “functional impairments” and it was completed both by the operator and the patient. It was decided that we use a declarative sentence with a binary response (True/False) for the first part of the questionnaire, while we used a rating scale with four point agreements (Bad, Satisfying, Good, Excellent) in the second part of the questionnaire. On the basis of the meticulous research of the available medical literature, 52 questions were designed. All of the questions were assessed and revised by a Croatian language expert.

A pilot study was conducted on a sample of 8 randomly chosen patients, and the feedback determined that the questions are clear and comprehensible. However, three questions were labeled as “too general” and were subsequently excluded from the questionnaire.

Hence, a questionnaire with 49 questions was administrated to the participants during the routine control check-up in the clinic. Average time needed for completing the questionnaire was 15 min. The patient–operator part was completed by a different operator than the one who conducted the “commando procedure” to avoid potential subjectivity and overvaluation of the results.

### 2.4. Statistical Analyses

All data analyses were performed using statistical software MedCalc (MedCalc Software, Ostend, Belgium, version 17.4.1). Quantitative data were expressed as mean ± standard deviation, while qualitative data were expressed as whole numbers. The Kolmogorov–Smirnov test was used to estimate the normality of the data distribution. Fisher’s exact test was used for the comparison of the qualitative data. The level of statistical significance was set at *p* < 0.05.

## 3. Results

### 3.1. Baseline Characteristics

The study included 34 patients (28 males and 6 females). The mean age of the participants was 58.2 ± 9.3 years. All of the patients underwent the “commando operation” and adjuvant radiotherapy. The mean time period from the “commando operation” was 3.6 ± 0.9 years, and the mean time period since the last radiotherapy session was 2.8 ± 0.8 years (Table 1). All of the patients received 66 Grays in 33 fractions over the course of the adjuvant radiotherapy.

Of the included patients, 28 underwent tracheostomy during the “commando operation”, and all of them were decannulated during the first 10 postoperative days. Furthermore, all patients had a nasogastric tube during the early postoperative period, and it was removed 20.3 ± 4.3 days from the operation.

All of the patients underwent partial glossectomy and en-block ipsilateral modified radical neck dissection with the preservation of n. accessorius. Additionally, 11 (32,3%) patients underwent marginal mandibulectomy. The surgical approach was the “pull through” procedure in 22 (64.7%) patients, while in 12 (35.2%) patients, the “lip split” technique was used.

### 3.2. Speech

Most of the patients (33, 97%) stated that their speech changed after the operation. Moreover, most of them (30, 88.2%) stated that they are understood over the phone and only two of them (5.8%) stated that they communicate only using written messages (Table 2).

Regarding the vowels, the most of them (23, 67.6%) have a problem with the pronunciation of the palatal consonants, while only four (11.7%) sought professional help (Table 2).

### 3.3. Eating Ability and Salivation Impairments

Most of the patients stated that they cannot chew (33, 97%) and swallow (33, 97%) as before the operation. All of them had problems with eating solid food, while none of them eat only liquid food or use the nasogastric tube. Moreover, only five (14.7%) sought professional advice and help (Table 3).

Most of the patients (32, 94.2%) stated that they have significant difference in salivation before and after the operation. Furthermore, most of them have a dry oral cavity (23, 67.6%) and trouble with a “thick” oral secretion (23, 67.6%) (Table 3).

### 3.4. Sleep Quality and Mood Changes

Almost half of the patients (16, 47%) have trouble with sleeping, while only seven (20.5%) of them use medication for sleeping. Furthermore, most of the patients (26, 76.4%) stated that they wake up several times during the night (Table 4).

Most of the patients (26, 76.4%) stated that they are prone to mood changes, and half of them (17, 50%) often feel discomfort (Table 4).

### 3.5. Aesthetic Results

Most of the patients (26, 76.4%) stated that they were aware of the possible aesthetic results, and 19 (55.8%) of them always see themselves the same no matter the operation. Only two (5.8%) patients would not undergo the operation today, no matter the consequences (Table 5).

### 3.6. Operator and Patient Subjective Assessment

There was a statistically significant difference between the operator and the patients in the subjective assessment of the aesthetic results (*p* = 0.047) as operators mostly assessed the results with grades “Good” (18, 52.9%) and “Excellent” (12, 35.2%), while the patients mostly assessed them with “Good” (17, 50%) and “Satisfying” (11, 32.3%) (Table 6).

There were no statistically significant differences between the operator and the patients in the subjective assessment of the functional impairments (*p* = 0.203) (Table 6).

Additionally, there were no differences in the patients’ outcomes between the two maxillofacial surgeons and between patients operated 1–3 years ago and those operated 4–5 years ago.

## 4. Discussion

The results of this study showed that most patients who underwent a composite operation of the head and neck due to the oral squamous cell carcinoma diagnosis had several functional impairments, and their quality of life was seriously impacted. Moreover, despite this severe impact on their functions, very few of them sought professional help regarding these issues. Additionally, our results showed that there were significant differences between the operators’ and patients’ subjective assessment about the aesthetic results as operators graded them significantly better than patients.

Speech is the main communication method in humans and, as such, plays a pivotal role in quality of life [32]. Most of our patients stated that their speech quality deteriorated after the operation, but on the other hand, most of them feel that their speech is intelligible over the phone to anyone. Additionally, only two patients stated that they communicate using written messages due to unintelligible speech. Regarding the production of speech sounds, most of the patients stated that they do not have a problem with the articulation of vowels, labiodental consonants, bilabial consonants, dental consonants, velar consonants, and liquid consonants. However, most of them had a problem with the articulation of the palatal consonants, which are especially complex in the Slavic languages. The palatal consonants are produced by the body of the tongue against the hard palate, and since these patients went through partial glossectomy and they have reduced mobility of the tongue, these results were anticipated but still interesting to show since palatal consonants are an important characteristic of the Croatian language.

Eating ability relies mainly on the chewing and swallowing activities, and both of these functions involve the tongue, which is an important mediator needed to properly accomplish them both [33,34]. Most of our patients stated that they cannot eat the same food as they used to before the operation. Moreover, all of them need great effort to eat solid food, and that is probably the reason why they prefer soft food. However, none of the patients eat only liquid food, and none of them needed a nasogastric tube since the postoperative period. Another interesting result is that most of the patients feel embarrassed eating in front of other people due to spontaneous gagging and coughing. Even though none of our patients underwent segmental mandibulectomy and they are not teethless, they still have trouble chewing solid food, and these results represent the importance of the tongue in the eating ability. However, the pectoralis major myocutaneus flap is a very thick flap that can create a bulk in the oral cavity that can possibly change the physiological movements needed to accomplish these functions. Our results are in alignment with the outcome of the study by Peleg et al. that also showed that all of the patients who underwent the pectoralis major myocutaneus flap reconstruction later reestablished functional swallowing [35]. Contrarily, the study by Fang et al. showed that only half of the patients had good swallowing capacities [36]. These discrepancies could be explained by the previously mentioned problems in conducting studies on patients who underwent head and neck surgery, such as great heterogeneity and a great number of variations in the procedure itself. Improvement of chewing and swallowing through rehabilitation should be a major goal after the “commando” operation. After using the modified barium swallow procedure to define the swallowing disorder, there are several maneuvers, postures, and exercises that can be used to treat and reduce eating impairments [37,38]. However, while there is some evidence that supports interventions aimed at improving swallowing and chewing, the efficiency of these different rehabilitation procedures still needs to be properly examined and evaluated [39].

Salivary problems are another set of problems that these patients experience after the commando operation, and they can have a large influence on their quality of life. Due to the vast excision of the oral mucosa, they experience oral dryness, while on the other hand, due to lower mobility of the tongue and lips, they have trouble keeping the saliva in their mouth. Most of our patients stated that they have a large difference in salivation before and after the operation. Furthermore, most of them feel embarrassed because of the drooling, while also most of them feel unpleasant due to the oral dryness and “thick” secretions that make it hard to cough. Even though all of our patients underwent unilateral submandibular gland excision as a part of the modified neck dissection, xerostomia is probably due to radiotherapy [40,41,42]. All of the included patients underwent adjuvant radiotherapy, which is a well-established cause of oral dryness that usually gradually improves [43]. However, xerostomia sometimes does irreparable damage to the salivary glands, and the improvement of the salivation output can be questionable.

Sleep quality is often taken as an indicator of a persons’ psychological state. Sleep disturbances are common in patients after surgery, but afterwards, sleep structure gradually returns to normal [44]. Still, sleep disturbances are associated with increased sensitivity to pain and higher cardiovascular risk [45]. Almost half of our patients stated that they have sleeping disturbances, and while most of them do not have problems with falling to sleep, almost all of them wake up several times during the night. Nevertheless, only a few of them use medication for sleeping, and only four of them were willing to seek professional help. A study conducted on patients with cancer in the oral cavity who underwent primary surgical resection showed that the prevalence of postoperative obstructive sleep apnea in these patients was a high 76% [46]. Since our patients, along with the intraoral resection, underwent modified radical neck dissection and reconstruction with the bulky the pectoralis major myocutaneus flap, it can be implicated that due to the changed anatomical and neuromuscular relations, they could possibly have a higher prevalence of obstructive sleep apnea. Moreover, recent systematic review results suggested that head and neck cancer patients have a higher incidence of obstructive sleep apnea when compared to the general population [47]. However, these results should be taken with caution since the exact etiology and subsequent correct management in these patients still needs to be addressed.

Even though the commando procedure produces a disfigurement of the facial aesthetics, most of our patients stated that they are satisfied with their appearance. Moreover, only two patients stated that they would not undergo the procedure if they knew what aesthetic consequences it would produce. Additionally, another important result that needs to be highlighted is the discrepancy between the operator and the patient in the subjective assessment of the aesthetic results. The operator evaluated the outcomes in almost all of the patients as “Great” or “Good”, while the patients mostly graded themselves with “Satisfying” and “Good”. Contrary to the aesthetic results, there was no significant difference in the evaluation of the functional impairments. These differences in the view of the aesthetic results could have been due to the different viewpoints of the operator and the patient. The operator in their everyday work sees a lot of aesthetic disfigurements, and according to their experience and knowledge, they are well aware of the possibilities of reducing these defects and the limits that could be accomplished. On the other hand, it seems that both the operator and the patients are more subjective in the assessment of the functional impairments.

There are several limitations in this study. First of all, it was administrated in a single center and designed as a case series. Furthermore, we were not able to eliminate all of the confounding effects such as survivorship and the time passed from the procedure, which could have possibly influenced the results. Moreover, even though we did homogenize our sample, as aforementioned, the commando operation and the oral squamous cell carcinoma are highly variable and there are still confounding factors that could not be completely eliminated. Our sample size was relatively small, and since we used a questionnaire as a tool to assess our parameters, there is a possibility that the patients overlooked, failed to recall, or had excess of subjectivity on some of the answers. Additionally, an assessment before the surgical procedure was not conducted; hence, our results could be biased by some pre-existing conditions and impairments. However, we believe that this was partly diminished with our strict inclusion and exclusion criteria.

## 5. Conclusions

In conclusion, the results of our study showed that patients who underwent the commando operation and the pectoralis major myocutaneus flap reconstruction due to the oral squamous cell carcinoma experience a large number of functional impairments, and the most prominently affected activities were speech, eating ability, and sleep quality. Moreover, we found a discrepancy between the operator and the patients in the assessment of the severity of the aesthetic disfigurements, while there was no difference regarding the assessment of the functional impairments. It is also important to highlight that even though most of the patients had severely impacted functions, almost none of them sought professional help. Lastly, there were no differences in the patients’ outcomes between two maxillofacial surgeons and between patients operated 1–3 years ago and those operated 4–5 years ago. These results are implying that this procedure and reconstructive method could possibly cause serious impairments for the patients’ wellbeing. Moreover, our outcomes also suggest that patients should be educated and rehabilitated after the “commando operation” since most of them were reluctant to seek professional help regarding their impairments. Lastly, sleep deficiency, which was noted among our subjects, could have an anatomical and pathophysiological background and it should be further explored.

## Figures and Tables

**Table 1:** Baseline characteristics of the study sample.

Parameter	*N* = 34
Male (N)	28
Female (N)	6
Age (years)	58.2 ± 9.3
Time since the operation (years)	3.6 ± 0.9
Time since radiotherapy (years)	2.8 ± 0.8
Dental status ^#^	
Healthy	6
Prosthesis	0
Bridge	13
Crowns of any type	20
Implants	12
Veneers	5
OSCC stage	
Stage III *	13
Stage IVA ^‡^	12
Stage IVB ^†^	9
OSCC location ^#^	
Mouth floor (sublingual)	16
Lateral tongue	7
Tongue base	6
Tonsillolingual	5
Tracheostomy (*N*)	
Yes	28
No	6
Partial glossectomy (*N*)	
Yes	34
No	0
Marginal mandibulectomy (*N*)	
Yes	11
No	23
“Pull through” method (*N*)	22
“Lip split” method (*N*)	12

All data are expressed as whole numbers or mean ± standard deviation. Abbreviation: OSCC—oral squamous cell carcinoma. * T2N1; ^‡^ T2N2, T3N2, and T4aN2; ^†^ T2N3, T3N3, T4aN3; ^#^ several of the patients had a combination of the stated conditions.

**Table 2:** Speech impairments of the study group.

Question	True	False
My speech is same as before the operation	1	33
I’m understood over the phone	30	4
Only my family and friends understand me	3	31
I mostly communicate using written messages	2	32
I have trouble speaking vowels E and I	2	32
I have trouble speaking vowels O and U	2	32
I have trouble speaking consonants B, P, and M	9	25
I have trouble speaking consonants D, T, N, C, Z, and S	13	21
I have trouble speaking consonants J, LJ, NJ, Č, Ć, DŽ, Đ, Ž, and Š	23	11
I have trouble speaking consonants K, G, and H	9	25
I have trouble speaking consonants L and R	10	24
I have trouble speaking consonants M and N	5	29
I use gesticulation more than before	3	31
I sought help from a professional speech therapist	4	30

All data are expressed as whole numbers.

**Table 3:** Eating and salivation impairments of the study group.

Question	True	False
I can chew as before the operation	1	33
I can swallow as before the operation	1	33
I have difficulty when eating solid food	34	0
I eat mostly soft solid food	33	1
I eat only liquid food	0	34
I eat only using the nasogastric tube	0	34
I don’t eat in front of other people due to gagging and coughing	31	3
I don’t eat in front of my family due to gagging and coughing	2	32
I sought advice from a professional regarding my diet	5	29
There is no difference in salivation before and after the operation	2	32
My oral cavity is dry	23	11
I have trouble with a “thick” oral secretion in the mouth	23	11
I feel embarrassed due to drooling	27	7
I’m drooling most of the time in a small amount	10	24
I’m drooling most of the time in a big amount	2	32
I avoid other people due to drooling	7	27

All data are expressed as whole numbers.

**Table 4:** Sleep impairments and mood changes of the study group.

Question	True	False
I have severe trouble with sleeping	16	18
I would like to sleep the whole day	2	32
I can’t fall asleep	10	24
I wake up during the night several times	26	8
I wake up early and can’t fall asleep again	10	24
I usually use medication for sleeping	7	27
I sought professional help regarding my poor quality of sleep	4	30
I’m prone to mood changes	26	8
My mood changes several times during the day	14	20
I often feel discomfort	17	17
I burst on the smallest annoyances	14	20
I often get depressed	10	24

All data are expressed as whole numbers.

**Table 5:** Aesthetic satisfaction of the study group.

Question	True	False
I was aware of the possible aesthetic results before the operation	26	8
I always see myself the same no matter the operation	19	15
Other people see me the same now and before the operation	15	19
I’m satisfied with my appearance	28	6
Today I would never undergo the operation no matter the consequences	2	32

All data are expressed as whole numbers.

**Table 6:** Operator and patient subjective assessment of the aesthetic results and functional impairments.

Aesthetic Results				Functional Impairments			
Grade	Operator	Patients	*p* *	Grade	Operator	Patients	*p* *
Bad	0	1	0.047	Bad	0	1	0.203
Satisfying	4	11		Satisfying	9	14	
Good	18	17		Good	19	17	
Excellent	12	5		Excellent	6	2	

All data are expressed as whole numbers. * Fisher’s exact test.

## Data Availability

All data is available from the corresponding author upon request.
